# Antitumour Activity of a pt(III) Derivative of 2-Mercaptopyrimidine

**DOI:** 10.1155/MBD.1997.9

**Published:** 1997

**Authors:** G. Cervantes, M. J. Prieto, V. Moreno

**Affiliations:** 1 Departament de Química Inorgànica Avgda Diagonal, 647 Barcelona E-08028 Spain; 2 Departament de Microbiología Universitat de Barcelona Avgda Diagonal, 645 Barcelona E-08028 Spain

## Abstract

The complex [Pt_2_Cl_2_(Spym)_4_], where Spym = 2-mercaptopyrimidine, was synthesized and analyzed spectroscopically. The presence in the ^195^Pt NMR spectrum, of only one signal for the Pt(III) indicates the symmetrical arrangement of the ligands and the identical setting of N, S and Cl atoms, PtS_2_ClN_2_, for the two Pt atoms being different to other compounds described in the literature. The interaction of this complex with DNA was studied by several techniques, including circular dichroism, melting temperature determination, electron microscopy (EM) and atomic force microscopy (TMAFM). Preliminary results show a high activity against HL-60 and HeLa tumour lines for the Pt-2-mercaptopyrimidine complex in comparison with *cisplatin* activity. Higher values for IC_50_ were obtained, while the values of LD_50_ were lower than those for *cisplatin*.


### ANTITUMOUR ACTIVITY OF A Pt(III) DERIVATIVE OF 2-MERCAPTOPYRIMIDINE

G. Cervantes[1], M. J. Prieto[2] and V. Moreno*[1]

[1] Departament de Química Inorgànica, Avgda Diagonal, 647 [2] Departament de Microbiología

Universitat de Barcelona, Avgda Diagonal, 645, E-08028 Barcelona, Spain

### Abstract

The complex $[Pt_2Cl_2(Spym)_4]$, where Spym = 2-mercaptopyrimidine, was synthesized and analyzed spectroscopically. The presence in the $^{195}Pt$ NMR spectrum, of only one signal for the Pt(III) indicates the symmetrical arrangement of the ligands and the identical setting of N, S and Cl atoms, $PtS_2ClN_2$, for the two Pt atoms being different to other compounds described in the literature. The interaction of this complex with DNA was studied by several techniques, including circular dichroism, melting temperature determination, electron microscopy (EM) and atomic force microscopy (TMAFM). Preliminary results show a high activity against HL-60 and HeLa tumour lines for the Pt-2-mercaptopyrimidine complex in comparison with *cisplatin* activity. Higher values for $IC_{50}$ were obtained, while the values of $LD_{50}$ were lower than those for *cisplatin.*

### Introduction

2-Mercaptopyrimidine is a potential model of biologically relevant molecules such as 2-thiouracil and 2-thiocytosine that are sometimes present in t-RNA, and it has also been tested as bacterial growth inhibitor and antiviral agent(1-3). However, until now, it has not been used as a drug. The development of the pyrimidine blues, polymeric compounds in which mixed-valence metal ions, usually platinum, are bridged by ligands derived from pyrimidines or amides, and the discovery of their high antitumour activity have extended the study to pyrimidine related-Pt(III) complexes. Several Pt(III) dimers have been described(4) and the structure of a dimer of Pt(III) with 2-mercaptopyrimidine was determined by Goodgame *et al.* (5).

There are several possibilities of coordination for the 2-mercaptopyrimidine molecule (Figure 1). It can bind to the metal as a neutral ligand *via* the S atom (A), or in bidentate fashion *via* N and S atoms (B), or as an anionic ligand (C), or finally as a bridging ligand to two metal atoms (D).

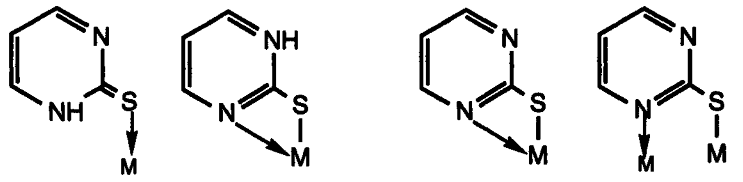

(A) (B) (C) (D)

**Figure 1**. Several coordination possibilities for 2-mercaptopyrimidine

Several compounds of 2-mercaptopyrimidine with various metals are described:

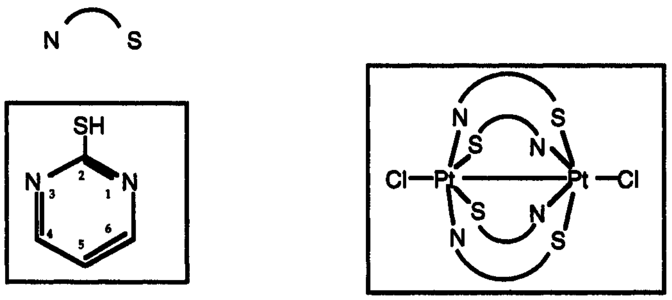

**Spym** **Pt-Spym**

**Figure 2**. Schematic representation of 2-mercaptopyrimidine and Pt-Spym

Abbot *et al.*[6] studied the complexes $M(Spym)_2$ (M=Co, Ni, Zn) with anionic and bidentate Spym; $M(Spym)_2X_2$ (M=Mn, Fe, Co, Ni and X=Br, Cl), (M=Zn and X=Cl) (M=Cd and X=Br, Cl and I) with anionic and bidentate Spym; Rosenfield *et al.*[7] described the complex $(Et_4N)[Ni(Spym)_3]$ with anionic and bidentate Spym; Taqui Khan *et al.*[8] found complexes of two types, [PdCl(Met)(Spym)]Cl with neutral and monodentate Spym, and $[Pd_2Cl_2(Spym)_2]$ with anionic and bidentate Spym; finally, Goodgame *et al.*[9] have synthesized the compounds $[Pt_2X_2(Spym)_4]$, (X=Cl, I) and $[Pt_2Cl_2(Spym)_5]$, where the oxidation state of platinum is III or a mixture of III and IV respectively. In several attempts to synthesize the mononuclear compounds of 2-mercaptopyrimidine and Pt(II) in 1:1 or 2:1 ratios, we always obtained the same compound $[Pt_2Cl_2(Spym)_4]$, identified as that studied crystallographically by Goodgame[5] (Figure 2).

Here we present a complete spectroscopic study together with a description of its effect on DNA structure and the results of several biological tests.

### EXPERIMENTAL

### *Materials and Methods*

The complex Pt-Spym was prepared using $K_2[PtCl_4]$ Johnson Matthey and 2-mercaptopyrimidine Aldrich. Elemental analyses were carried out on a Carlo Erba 1500 microanalyzer at the Serveis Científico-Tècnics at the University of Barcelona. The infrared spectra were recorded in solid state (KBr pellets) on an FT-IR Nicolet 5DZ spectrometer in the 4000-400 $cm^{-1}$ range. $^{1}H\{^{13}C\}$, $^{13}C\{^{1}H\}$ and $^{195}Pt\{^{1}H\}$NMR spectra were obtained on a Brucker DRX 250 spectrometer using DMSO-$d_6$ as solvent. Chemical shifts were measured relative to TMS in the case of $^{1}H$ and $^{13}C$ and to $Na_2PtCl_6$ for $^{195}Pt$.

UV spectra were recorded at room temperature on a double beam SHIMAZDU UV-2101-PC spectrometer. The absorbance change during DNA denaturalization (Tm) was measured on the same SHIMAZDU UV-2101-PC spectrometer with thermostatic cells connected to an automatic temperature regulator NESLAB RTE-110 bath. The heating rate was 1.5 ºC/min. The absorbance was measured at $\lambda$ = 260 nm. CD spectra were obtained on a JASCO J720 spectropolarimeter with a 450 W xenon lamp.

Samples, prepared as described below, were observed on transmission electronic microscopes Philips EM 200 and Philips EM 301 at 80,000 V, the first working at 25,800 and 54,900 magnification and the second at 16,000, 20,000 and 26,000 magnification. Over 82.5 ml TAE, 25 ml 0.1% cytochrome c, 25 ml 38% formaldehyde and 610 ml pure water solution, the adduct PBR322:Pt-Spym with $r_i$ = 0.50 and 72 h of incubation was added. Microdrops were prepared from this solution and after 20 min Ni MESH200 grills recovered with FORMVAR membrane and reinforced with graphite were deposited over DNA drops. The samples were shaded by evaporation of $WO_3$ in an evaporation chamber at $5.10^{-6}$ torr, under nitrogen atmosphere for 10 min by a 20 A and 150 W current with a rotation of 100 rpm.

Images of linear DNA, 200 base-pair copy of *Escherichia coli,* and adducts of Pt compounds and this DNA were obtained with Extended Nanoscope III (Digital Instruments, Santa Barbara, CA) working in TMAFM mode in about 100 nN. The samples were prepared as described above and 2 ml of each was deposited on a small mica disk, washed in distilled water after 20 min, and dried under argon.

*In vitro* studies, cytotoxicity in tumour lines and type of cell death were determined in Centro de Biologia Molecular "Severo Ochoa" at Universidad Autónoma de Madrid-CSIC.

### *Synthesis of the Complex*

1 mmol of $K_2PtCl_4$ was dissolved in 10 ml of water and mixed with a suspension of 1 mmol of 2-mercaptopyrimidine (Spym) in 10 ml of hot water. After a few minutes a pale brown precipitate was formed and after stirring for 3 h at 40 ºC and 48 h at room temperature a dark brown solid appeared. The product was filtered off the orange solution, washed with $H_2O$ and ethanol and finally dried over silica gel. Calculated for $[Pt_2Cl_2(Spym)_4]$ C, 21.18; N, 12.35; H, 1.55; S, 14.12; Cl, 7.81. Found: C, 21.42; N, 12.07; H, 1.60; S, 13.84; Cl, 7.87.

When the synthesis was repeated using the ratio $K_2PtCl_4$ : Spym 1:2, the same product was obtained while the solution remained colourless.

### RESULTS AND DISCUSSION

### *FT-IR Spectra*

The IR data for 2-mercaptopyrimidine and the platinum complex are shown in Table 1.

The band at 1217 $cm^{-1}$ was assigned to $\nu_{st}$(SH) of the thiol form, and the band at 1188 $cm^{-1}$ .was assigned to $\nu$(NH) and $\nu$(C=S) of the thiona form.

Table 1: Main IR frequencies ($cm^{-1}$) of 2-mercaptopyrimidine and Pt-Spym

| ASSIGNMENT | Spym | Pt-Spym |
|---|---|---|
| $\nu_{st}$(SH) | 2618,2521 | — |
| ν(NH) | 2841,2730 | — |
| ν(C=C)+ν(C=N) | 1609 vs<br>1570 s | 1605s<br>1575s<br>1542m |
| $\nu(C\text{-}S)_{thiol}+\delta(ring)$ | 1217m | 1248m |
| $\nu(C{=}S)_{thiona}+\delta(ring)$ | 1188s | 1175m |
| ν(C=N)+ν(C=S)<br>(thioamida III) | 983m | 1026w<br>1024w |
| $\gamma(CS)_{out\ plane}+\gamma(CH)$<br>(thioamida IV) | 746m | 752s |
| γ(C-S)+τ(ring)out plane | 471m | 484m |
| ν(Pt-S) | | 440,432w |
| ν(Pt-Cl) | | 296w |
| δ(Pt-N) | | 225w |

s: strong, m: medium, w: weak

These results indicate that the two tautomeric thiol and thiona forms are present in the solid [3,10-12]. Three weak bands at 2063, 1977 and 1915 $cm^{-1}$ assigned to aromatic ring combination bands disappeared when the complex was formed due to the electronic changes induced by the coordination of the metal ion. The band at 1188 $cm^{-1}$ assigned to ν(C=S) of the thiona form [11,13-14] in 2-mercaptopyrimidine decreased in frequency and intensity in the complex indicating the coordination through the S atom [15]. In contrast, the band at 1217 $cm^{-1}$ corresponding to ν(C-S) of the thiol form increased in frequency when the metal ion bound to 2-mercaptopyrimidine. The other bands related with S bonds also showed changes in frequency and intensity. Special attention should be given to the thioamide bands resulting in the coupling between different vibrations when a C=S group is bound to one or more N atoms [15]. The band assigned to thioamide III (ν(C=N) + ν(C=S) ) at 983 $cm^{-1}$ split and increased in frequency in the complex $[Pt_2Cl_2(Spym)_4]$, as has been observed for bidentate (N,S) compounds. On the other hand, the $\nu_{st}$(SH) bands disappeared, indicating that the thiol form is not present when the metal ion is coordinated by sulphur. A new band corresponding to ν(Pt-S)[8, 16-18] was also observed for the complex. The splitting of the band assigned to ν(C=C) + ν(C=N) at 1570 $cm^{-1}$ in the Pt compound indicates that the pyrimidine ring may be involved in the coordination to the metal. The appearance of a new band at 225 $cm^{-1}$ assigned to δ(M-N) [8,19, 20] and the behaviour of the thioamide III band seem to reinforce this hypothesis. A new band at 296 $cm^{-1}$ can also be observed. Although the bands corresponding to terminal ν(Pt-Cl) in Pt(II) compounds appeared at frequencies between 345 and 310 $cm^{-1}$[9,21,22], the Pt(III) dimers with terminal Cl showed the ν(Pt-Cl) absorption at lower frequencies[23,24]. This shifting is due to the lengthening of the Pt-Cl bond caused by the *trans* effect of the Pt-Pt bond of the dimer[5,25-27]. So, the FT-IR data are consistent with the coordination of the four 2-mercaptopyrimidine molecules by both sulfur and nitrogen atoms bridging the two Pt(III) atoms. Each metal atom is bound to one terminal Cl atom.

### *$^{1}H$, $^{13}C$ and $^{195}Pt$ NMR Study*

The main chemical shifts assigned in the $^{1}H$ NMR spectrum of the 2-mercaptopyrimidine and the compound $[Pt_2Cl_2(Spym)_4]$ are shown in Table 2. In the spectrum of the free 2-mercaptopyrimidine, a complex pattern appears at low frequency, corresponding to H(5) coupled with the neighbouring protons. The signal observed at 8.29 ppm is assigned to H(4) and H(6). The presence of a broad signal at 12.85 ppm, assigned to NH proton, together with the observed equivalence of H(4) and H(6) suggests a fast exchange of the NH proton between sites 1 and 3. The major form in DMSO is the thiona form[28]. Several changes can be observed in the Pt complex spectrum in comparison to that of the free 2-mercaptopyrimidine. The H(4) and H(6) are no longer equivalent. The signal corresponding to NH disappeared, indicating that the ligand is deprotonated and that it coordinates in bidentate fashion *via* S and N atoms. The large downfield shift (0.95 ppm) observed for H(4) suggests that N(3) is involved in the coordination. The signal assigned to H(5) appeared as a triplet with a J value of 4.9 Hz, as expected for an *ortho* coupling. The H(4) and H(5) are coupled with J=4.4 Hz, which is in agreement with values reported elswhere for this type of ligand. The signal assigned to H(6) is not well resolved and it is not possible to determine the J

value. In the case of other Pt(III) complexes similar $^1H$ NMR signals were observed [(25)]. Although the complex can react with DMSO and the Pt-Pt bond can be broken easily to give monomeric species, no solvolysis signal was obseved. The low- temperature (246K) spectrum is the same as that recorded at room temperature.

Table 2: Chemical shifts (ppm) from $^1H$ NMR$^a$ spectrum of 2-mercaptopyrimidine and Pt-Spym

| Assignment | NH | H4 | H6 | H5 |
|---|---|---|---|---|
| Spym | 12.85w | 8.29s | 8.29s | 6.86m |
| Pt-Spym | b | 9.24d | 8.68s | 7.36t |

a : registered in DMSO at room T ( intern reference:TMS)
b: not observed m:complex pattern d:double t:triplet w:wide

In Table 3 the $^{13}C$ shifts for 2-mercaptopyrimidine and the compound $[Pt_2Cl_2(Spym)_4]$ are presented.

Table 3: Chemical shifts (ppm) in $^{13}C$ NMR$^a$ spectrum of 2-mercaptopyrimidine and Pt-Spym

| Assignment | C2 | C4 | C6 | C5 |
|---|---|---|---|---|
| Spym | 181.97 | 154.37$^w$ | 154.37$^w$ | 109.42 |
| Pt-Spym | 180.69 | 158.63 | 157.36 | 117.76 |

w: wide a : in DMSO at room T (internal reference:TMS)

In the complex, the equivalence between C(4) and C(6) signals is lost. The most noticeable shift corresponds to C(5), followed by C(4) and C(6). The quaternary C(2) appears as a very weak signal showing only an insignificant shift due to the low sensitivity of carbon atoms of this kind. The large shift of C(5) can be explained because this atom is in *para* with respect to the S atom coordinated to the Pt[(29)]. The N coordinated to the metal atom is probably the closest to C(4) because the corresponding signal shifts more than that assigned to C(6).

Finally, the $^{195}Pt$ spectrum for the complex $[Pt_2Cl_2(Spym)_4]$, shows a single signal at -1179 ppm, related to $K_2PtCl_6$, which represents an intermediate shift between those of Pt(II) and Pt(IV) for a similar environment, and which corresponds to an oxidation state of Pt(III)[(27,30,31)].

The presence of only one signal for the Pt(III) indicates the symmetrical arrangement of the ligands and the identical setting of N, S and Cl atoms, $PtS_2ClN_2$, for the two Pt atoms. In the case of the analogous compound described by Goodgame[(5)] the crystal structure indicates that two types of Pt environment, $PtSClN_3$ and $PtS_3ClN$ may be present. The different chemical environment must lead to the appearance of two signals in the $^{195}Pt$ spectrum but the authors did not reported anything about it. However, the equivalent dimer compound with iodine $[Pt_2I_2(Spym)_4]$, also studied crystallographically by Goodgame[(9a)], presents the same chemical environment $PtS_2IN_2$ for both Pt.

### *Circular Dichroism*

The circular dichroism spectra of *Calf Thymus* DNA and *Calf Thymus* DNA incubated with the mercaptopyrimidine-Pt compound at several times and with several molar ratios were registered. In Tables 4a, 4b and 4c, the $\Theta_{max}$ and $\Theta_{min}$ for $\lambda_{max}$ and $\lambda_{min}$ values at different $r_i$ and incubation times, can be observed.

Table 4a: CD of DNA:Pt-Spym complex at different molar ratios (incubation time16 h )

| $r_i$ | $\Theta_{max}{}^a$ | $\lambda_{max}{}^b$ | $\Theta_{min}$ | $\lambda_{min}$ |
|---|---|---|---|---|
| DNA | 8.78 | 276.3 | -9.77 | 245.6 |
| 0.01 | 8.48 | 277.8 | -9.83 | 245.7 |
| 0.10 | 8.88 | 275.8 | -9.95 | 244.6 |
| 0.25 | 8.24 | 271.1 | -11.61 | 241.5 |
| 0.50 | 7.92 | 277.7 | -8.84 | 246.0 |

a: $^oC.cm^2.dmol^{-1}.10^3$ b: nm

The major changes occur for $r_i = 0.25$. There was a slight modification of DNA spectrum when the Pt-Spym complex was bound to the helix. This change was more marked at 16 h of incubation. The positive band has less ellipticity than the DNA one and this effect, together with the increase of the ellipticity and the decrease of the wavelength, indicate changes in the base stacking, probably due to the opening of the helix[32]. The behaviour is very similar to that induced by *transplatin* , which originates helix opening due to the binding between the two strains of the same DNA helix. The structure of the Pt-Spym compound that presents two labile sites (Cl) at the ends of the dimer can allow a similar type of binding.

Table 4b: CD of DNA:Pt-Spym at different molar ratios (incubation time 48 h )

| $r_i$ | $\Theta_{max}{}^a$ | $\lambda_{max}{}^b$ | $\Theta_{min}$ | $\lambda_{min}$ |
|---|---|---|---|---|
| DNA | 8.61 | 276.0 | -9.34 | 245.0 |
| 0.01 | 8.36 | 277.2 | -9.07 | 245.6 |
| 0.10 | 8.82 | 274.2 | -9.48 | 244.6 |
| 0.25 | 8.93 | 275.4 | -9.55 | 245.8 |
| 0.50 | 8.53 | 276.2 | -9.22 | 246.4 |

a: $^{\circ}C.cm^2.dmol^{-1}.10^3$ b: nm

Table 4c: CD of DNA:Pt-Spym at different times ($r_i$=0.25)

| $t^c$ | $\Theta_{max}{}^a$ | $\lambda_{max}{}^b$ | $\Theta_{min}$ | $\lambda_{min}$ |
|---|---|---|---|---|
| DNA | 8.48 | 277.0 | -9.83 | 245.6 |
| 16 | 8.24 | 271.1 | -11.61 | 241.5 |
| 24 | 7.94 | 276.6 | -12.22 | 241.2 |
| 48 | 8.93 | 275.4 | -9.55 | 245.8 |

a: $^{\circ}C.cm^2.dmol^{-1}.10^3$ b: nm c: h

However, after 48 h of incubation, the changes are less marked, although the greatest alteration occurs also for $r_i$ = 0.25. In this case the main change is the stabilization of the double helix and the increased coiling. Probably, the long time of incubation produces the disappearance of the interaction observed at 16 h.

The ellipticity of the positive band decreases when compared to that of DNA at 16 h and 24 h and increases slightly at 48 h. In contrast, the ellipticity of the negative band always increases and reaches the major change at 24 h for $r_i$ = 0.25. The type of interaction observed at 16 h remains until 24 h which is when the major change occurs. It is probable that the adduct formed, DNA:Pt-Spym, remains only until 24 h.

### *UV . Melting Temperature (Tm)*

The wavelengths and the absorbances corresponding to the maximum UV of the complex DNA:Pt(Spym) are collected in Table 5.

Table 5: UV wave lenghts and maximum absorbance$^a$ of DNA:Pt-Spym complex at different $r_i$ (16 and 18 h)

| COMPLEX | ri | 16 h | | 48 h | |
|---|---|---|---|---|---|
| | | $\lambda_{max}{}^b$ | Abs | $\lambda_{max}{}^b$ | Abs |
| DNA | | 258.6 | 0.365 | 258.1 | 0.373 |
| DNA:Pt-Spym | 0.01 | 258.3 | 0.368 | 258.9 | 0.369 |
| DNA:Pt-Spym | 0.10 | 258.3 | 0.382 | 257.5 | 0.383 |
| DNA:Pt-Spym | 0.25 | 257.3 | 0.388 | 258.0 | 0.400 |
| DNA:Pt-Spym | 0.50 | 257.2 | 0.471 | 256.5 | 0.587 |

a: (Abs DNA:complex - Abs complex) at 25 °C b: nm

The percentages of hyperchromicity (positive value) or hypochromicity (negative value) at 260 nm and the $\Delta A_{270}/\Delta A_{295}$ ratio are shown in Table 6.

In the adduct DNA:Pt-Spym a strong shifting of the maximum towards lower wavelenghts was observed. This effect is greater for higher $r_i$ values (0.25, 0.50) and a progressive increase in the hyperchromicity when the Pt/nucleotide ratio increased was also observed. This seems to indicate a strong modification of the secondary DNA structure. Going from $r_i$ = 0.25 to $r_i$ = 0.50 a a b

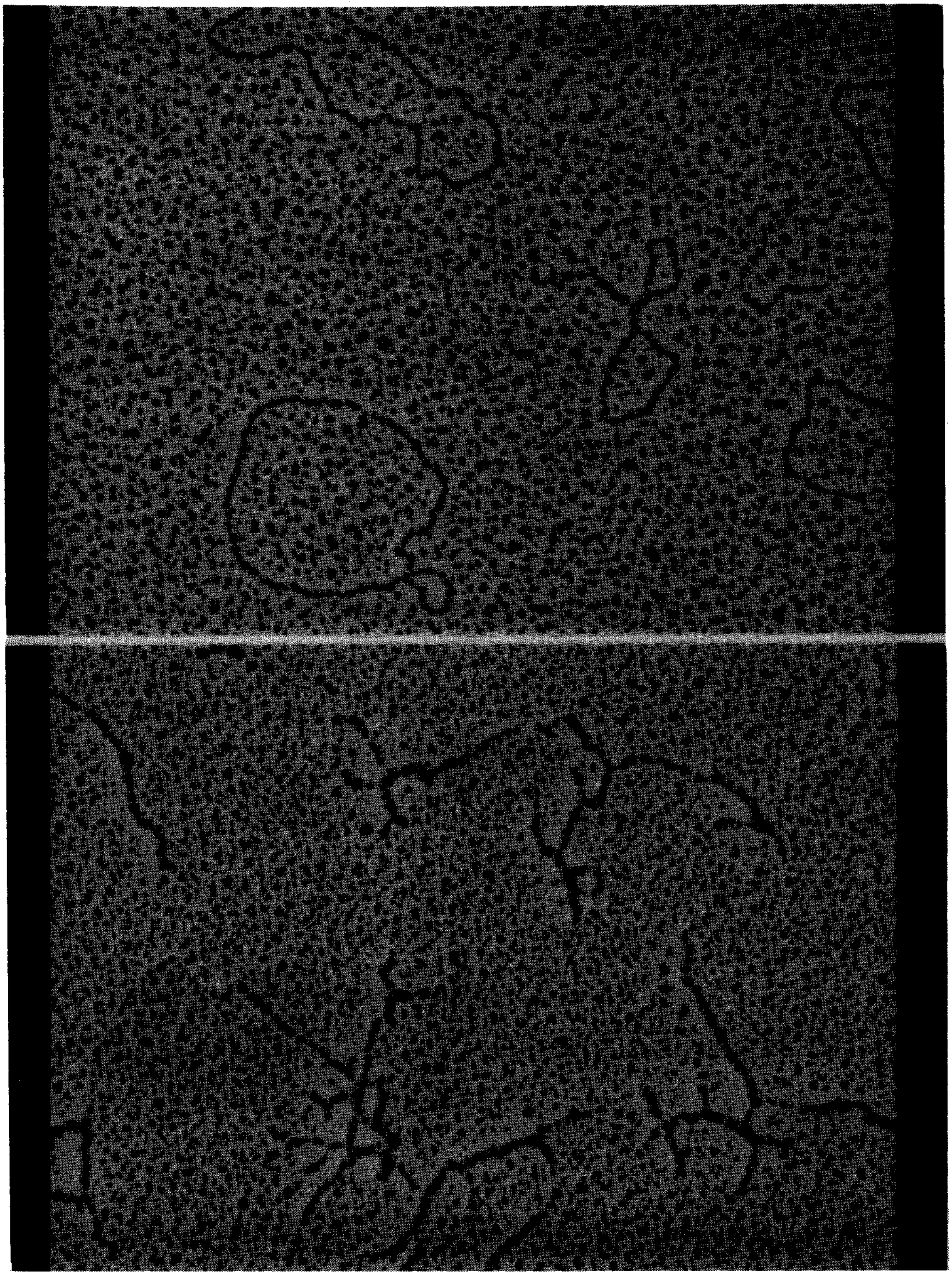

**Figure 3.** Microphotographs corresponding to plasmid alone (a), and to plasmid incubated for 24 h. with Pt-Spym at $r_i = 0.50$ (b)

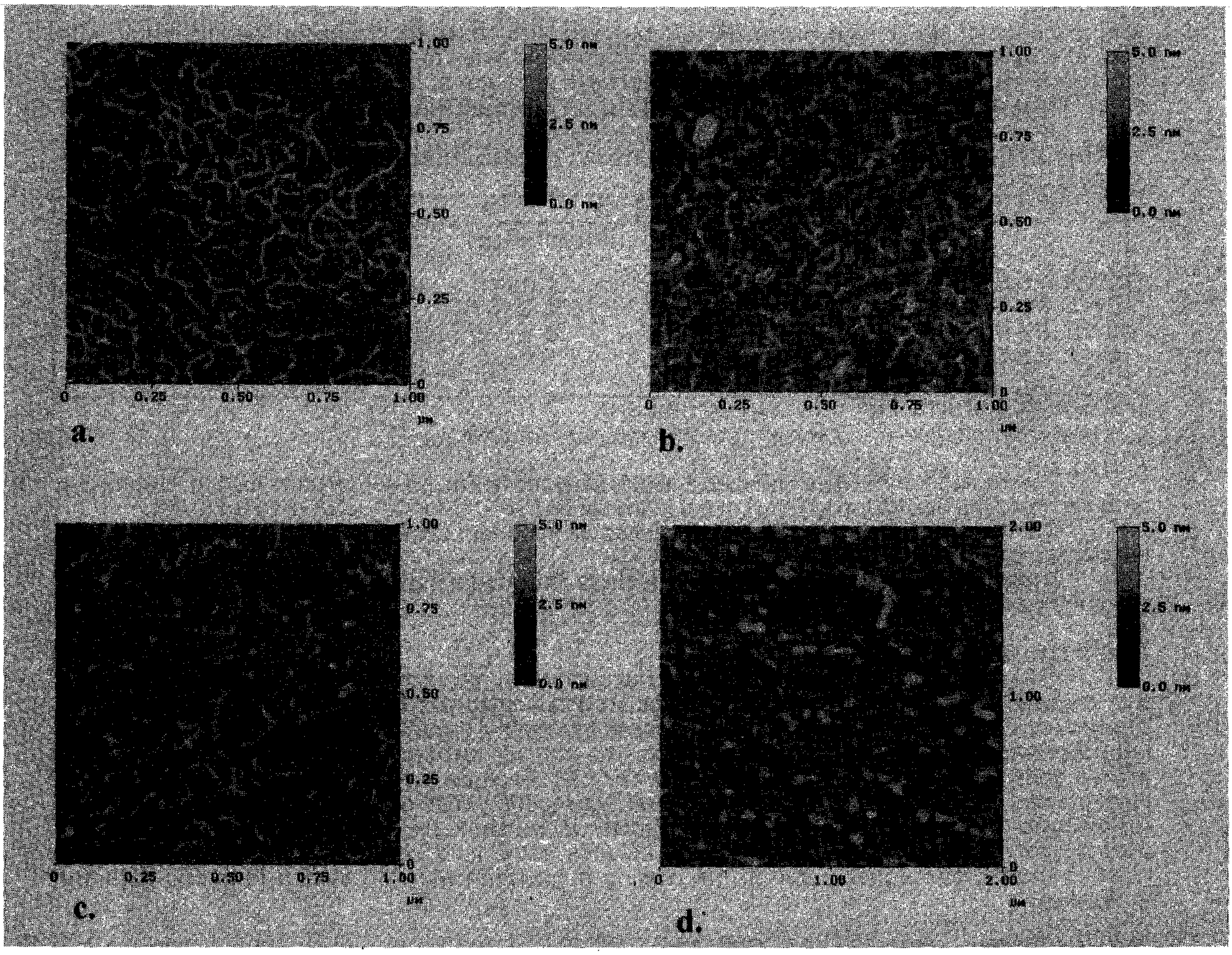

**Figure 4.** TMAFM images corresponding to linear fragments of DNA, hliM (a), to the same DNA incubated for 24 h. with cisplatin (b), incubated for 24 h. with Pt-Spym (c), and incubated for 24 h. with Spym alone (d)

strong change of percentage H and $\Delta A_{270}/\Delta A_{295}$ was also observed. Probably, the interaction with DNA of a bulk compound with two weak sites on opposite sides of the complex causes uncoiling of the double helix and destabilizes the secondary DNA structure at room temperature. The values of $\Delta A_{270}/\Delta A_{295}$ indicate that the Pt compound interacted with the DNA.

Table 6: % hyperchromicity[a] (+) or hypochromicity(-) at 260 nm and $\Delta A_{270}/\Delta A_{295}$[b] ratio in DNA:Pt-Spym complex at different ri at 16 and 48 h

| COMPLEX | ri | 16 h | | 48 h | |
|---|---|---|---|---|---|
| | | %H | $\Delta A_{270}/\Delta A_{295}$ | %H | $\Delta A_{270}/\Delta A_{295}$ |
| DNA:Pt-Spym | 0.01 | +1.1 | 1.0 | -0.4 | 1.7 |
| DNA:Pt-Spym | 0.10 | +4.7 | 1.6 | +2.4 | 2.0 |
| DNA:Pt-Spym | 0.25 | +5.8 | 0.4 | +6.7 | 3.3 |
| DNA:Pt-Spym | 0.50 | +27.1 | 7.0 | +53.4 | 2.2 |

a: %H = (Abs DNA:complex - Abs DNA)100 / Abs DNA

b: $(\text{Abs DNA:complex - Abs DNA})_{270}$ / $(\text{Abs DNA:complex - Abs DNA})_{295}$

The compound Pt-Spym induces a slight decrease in Tm. In compounds having intercalator aromatic rings the values found for ΔTm are in the range 0.4-1 ºC for CT- DNA at pH= 7.0 (33).

On the other hand, this compound caused an important decrease in the absorbance at high temperatures.

These results together with the UV spectrum indicated a marked interaction with the DNA at room temperature.

### *Electron Microscopy*

In Figure 3, the microphotographs corresponding to plasmid alone(a), to plasmid incubated with *cisplatin* at $r_i$ = 0.50 for 24 h (b), and to plasmid incubated with Pt-Spym at $r_i$ = 0.50 for 24 h (c) are shown. The plasmid alone is in circular closed form with different degrees of supercoiling, and a low percentage of linear form can also be seen. The *cisplatin* causes a compactation in the plasmid, as has been described in the literarure (34) which can reach up to 50 %. The Pt-Spym compound also causes a compactation, but also lateral aggregation with other plasmid molecules. This compactation is more intense than that of *cisplatin* in the same experimental conditions. This fact may indicate that the Pt-Spym compound induces some kind of interaction between different plasmid molecules, probably due to the binding of platinum with two strains from two different molecules.

### *Atomic Force Microscopy (TMAFM)*

Figure 4 shows the images corresponding to linear fragment DNA, *hliM* (a), the same DNA incubated 24 h with *cisplatin* (b), incubated 24 h with Pt-2-mercaptopyrimidine (c), and 24 h with 2-mercaptopyrimidine alone (d). The concentration in DNA is the same in all the samples. *Cisplatin* seems to compact and distort. In the magnification corresponding to 400 nm, several points where the fragment is doubled can be observed. The measurement of the length of this fragment on the microscope reveals this modification. The measurement of the area also shows that there is no aggregation between different linear DNA fragments. So, the binding type of *cisplatin* to DNA, mainly to two N intra-strain sites, is reflected. However, after DNA incubation with Pt-Spym, always in the same conditions as for *cisplatin*, aggregation and compacting phenomema were observed. The aggregate formed have larger area than a single fragment, indicating that the presence of the complex has produced the aggregation of several DNA fragments. So, the binding of Pt-Spym induces an interaction between different DNA strains. Probably, it binds through the two lateral sites after Pt-Cl has been hydrolyzed. The effect observed is not due to the ligand. The effect on the DNA in the same conditions as for 2-mercaptopyrimidine alone can be observed in Figure 4(c). The measurement of the length of the strain on the microscope confirms that neither lengthening or distorsion has been caused.

### *Preliminary Cytotoxicity tests on Tumour lines*

The tests have been carried out on two tumour lines: HeLa cells from an uterus cancer and HL-60 cells from human leukemia. The trials were performed with *cisplatin* as reference, and with the Pt-2-mercaptopyrimidine complex. The $LC_{50}$ parameters are summarized in Tables 7 and 8.

The values for the Pt-Spym are better than for the *cisplatin* against the tumour line HL-60. The percentage of cell death is high at very low concentrations of complex, 3.5 mM, which is markedly lower than the one obtained for *cisplatin*.

Table 7: $LC_{50}$ for *cisplatin* and Pt-Spym in the tumour line HL-60

| Compound | $LC_{50}$(μM) | $LC_{50}$(μg/ml) |
|---|---|---|
| **cisplatin** | 35.0 | 10.5 |
| **Pt-Spym** | 3.5 | 3.3 |

Table 8: $LC_{50}$ for *cisplatin* and Pt-Spym in the tumour line HeLa

| Compound | $LC_{50}$(μM) | $LC_{50}$(μg/ml) |
|---|---|---|
| **cisplatin** | 37.0 | 11.1 |
| **Pt-Spym** | 3.5 | 3.3 |

The difference between *cisplatin* and the Pt-Spym compound is still greater for the tumour line HeLA. Although the $LC_{50}$ value for Pt-Spym is the same, cell death is practically complete at 5 mM. For *cisplatin,* the same effect was produced only at concentrations near 150 mM. 2-Mercaptopyrimidine alone did not produce cell death. Probably the dramatic effects of the platinum complex over DNA are related with the structure of the complex but the possible role of the oxidation state III of the platinum in the mechanisms of cytotoxicity cannot be ruled out[(35)].

The analysis of the type of cell death was carried out with two kinds of tests: the observation of the characteristic morphological changes by means of phase contrast microscopy and the checking of DNA digestion in regular fragments by gel electrophoresis. This exposure in HeLA cells, at 16 or 24 h, produced the characteristic changes due to apoptosis.

The same test was carried out with the tumour line HL-60. The Pt-Spym does not induce apoptosis in this tumour line.

*In vivo* toxicity test with male mouses $BDF_1$ gave a $DL_{50}$ value for the Pt-Spym compound of 250 mg/Kg. This value indicates that the toxicity is much lower than those of *cisplatin* or *carboplatin,* which are the first and second generation platinum complexes used in clinical trials.

### ACKNOWLEDGMENTS

*We are grateful to DGICYT Ref. PB91-0806 and to Euroepan Community HCM, ref. ERBCHRXCT 920016 for financial support, to Johnson Matthey for $K_2PtCl_4$ supplied, and C Alonso and VM Gonzalez from Centro de Biologia Molecular, "Severo Ochoa", UAM, Cantoblanco, Madrid, for biological tests.*

### REFERENCES

1. Gutierrez-Valero M.D., Romero-Molina M., L6pez-Garz6n R., Salas-Peregrfn J.M.,*Trans. Met. Chem.*,1988,**13**, 451.
2. Holy A., Votruba I., Jost K., *FEBS Lett.* ,1972, **22**, 287.
3. Nowak M., Rostkowska H., Lapinski L., Leszczynski J., Kwiatkowskil J., *Spec. Acta* , 1991, **47A**,339.
4. Lippert B., Arpalahti J., Krizanovic O., Micklitz W., Schwarz F., Trötscher G. in Nicolini M. (ed) 1987, Martinus Nijhoff Publishing, Boston
5. Goodgame D.M.L., Rollins R.W., Slawn A.M., Williams D.J., Zard PW; *Inorg. Chim. Acta* ,1986,**120**, 91
6. Abbot J., Goodgame D.M.L., Jeeves I.,*J. Chem. Soc. Dalton* ,1978, 880.
7. Rosenfield S.G., Berends H.P., Gelmini L., Stephan D.W., Mascharak P.K., *Inorg. Chem.* ,1987, **26**, 2792.
8. Taqui Khan B., Bhatt J., Najmuddin K., Shamsuddin S., Annapoorna K., *J. Inorg. Biochem.* ,1991, **44**, 55.
9. a) Goodgame D.M.L., Rollins R.W., Skapski A.C. *Inorg. Chim. Acta* ,1984, **83** L11 b) Goodgame D.M.L., Slawn A.M., Williams D.J., Zard P.W., *Inorg. Chim. Acta* ,1988, **148**, 5
10. Battistuzzi R., Petronel G., *Spectrochim. Acta* ,1980),**36A**, 113
11. Goel R., Gupta S., Sharma S., Gupta C., *J.Chem.Soc.Faraday Trans.II* ,1986, **82**, 123
12. Contreras G., Seguel G., Alderete J., *Spectrochim. Acta* ,1994),**50A**, 371
13. Spinner E., *J.Chem.Soc.* ,1960,1237
14. Kennedy B.P., Lever A.B.P., *Can. J.Chem.* ,1972,**50**, 3488
15. Raper E.S., *Coord.Chem.Rev.* ,1985),**61**. 115
16. Ginn V.C., Kelly P.F., Slawin A.M., Williams D.J., Woollins J.D., *J.Chem.Soc.Dalton* ,1992),963
17. Akovidis A., Hadjiliadis N., *Coord.Chem.Rev.* ,1994, **135**, 17
18. De Sousa G.F., Filgueiras C.H., *Trans.Met. Chem.* ,1990),**15**, 290
19. Adams D.M.,1967, *Metal-Ligand and Related Vibrations*, St Martin's Press, New York.

20. Mikulski C., De Prince R., Tran T., *Inorg. Chim. Acta* ,1981, **56**, 27
21. Nakamoto K., 1986, *Infrared and Raman Spectra of Inorganic and Coordination Compounds,* 4th ed, Wiley Interscience, Chichester
22. Berg R., Rasmussen K., *Spectrochim. Acta* ,1973),**29A**, 319
23. Stein P., Dickson M., Roundhill D.,*J.Am.Chem.Soc.* ,1983, **105**, 3489
24. Che C., Schaefer W., Gray H., Dickson M., Stein P., Roundhill D., *J.Am.Chem.Soc.* ,1982, **104**, 4253
25. Lippert B., Schollholm H., Thewald U., *Inorg. Chem.* ,1986, **25**, 407.
26. Alexander K., Bryan S., Fronczek F., Fultz W., Rheingold A., Roundhill D., Stein P., Watkins S., *Inorg. Chem.* ,1985, **24**, 2803
27. Woollins J.D., Kelly P.F. *Coord.Chem.Rev.* ,1985, **65**, 115
28. Chenon M., Pugmire R., Grant D.M., Panzica R., Townsend L., *J.Am.Chem.Soc.* ,1975),**97**, 4627
29. Seddon K., Turp J., Constable E., Wernberg O., *J.Chem.Soc.Dalton* ,1987, 293
30. Pregosin P. (ed), 1991 ,*Transition Metal Nuclear Magnetic Resonance*, Elsevier, Zürich.
31. Wienkötter T., Sabat M., Fusch G., Lippert B., *Inorg. Chem.* ,1995),**34**, 1022
32. Johnson A., Qu Y., Van Houten B., Farrell N., *Nucl. Acid Res.* ,1992, **20**, 1697
33. McConnaughie A., Jenkins T., *J.Med.Chem.* ,1995),**38**, 3488
34. Keck M., Lippard S.J.,*J.Am.Chem.Soc.* ,1992, **114**, 3386
35. Farrell N. (ed) ,1989, *Transition Metal Complexes as Drugs and Chemotherapeutic Agents,* Kluwer Academic Publishers, Dordrecht
36. Lowe S.W., Ruley H.E., Jacks T., Housman D.E.,*Cell* ,1993,**74**, 957

**Received: January 3, 1997 - Accepted: January 21, 1997 -**
**Received in revised camera-ready format: January 23, 1997**

